# Challenges in the Implementation of Competency-Based Medical Curriculum: Perspectives of Prospective Academicians

**DOI:** 10.7759/cureus.32838

**Published:** 2022-12-22

**Authors:** Rashmi Ramanathan, Jeevithan Shanmugam, Sridhar M Gopalakrishnan, Kalaniti T Palanisamy, Seetharaman Narayanan

**Affiliations:** 1 Physiology, Kovai Medical Center and Hospital (KMCH) Institute of Health Sciences and Research, Coimbatore, IND; 2 Community Medicine, Kovai Medical Center and Hospital (KMCH) Institute of Health Sciences and Research, Coimbatore, IND; 3 Biochemistry, Kovai Medical Center and Hospital (KMCH) Institute of Health Sciences and Research, Coimbatore, IND; 4 Medicine, Kovai Medical Center and Hospital (KMCH) Institute of Health Sciences and Research, Coimbatore, IND

**Keywords:** revised basic course workshop, new perspective, curriculum implementation support programme (cisp), faculty development program, competency based medical education (cbme)

## Abstract

Introduction

The medical education system all over the world is witnessing a paradigm shift from traditional methods of teaching to competency-based medical education. With the current curricular change, teachers are supposed to play a catalyst role in terms of moderating the different frameworks of competency-based medical education (CBME). Following the implementation of the new curriculum in India (2019), the present study aims to understand the challenges that medical teachers face in its implementation.

Materials and methods

This nationwide cross-sectional study was conducted among 297 teaching faculty representing 91 medical colleges across 20 states between February and July 2020. A self-validated structured questionnaire on the views of the newly implemented competency-based medical curriculum was prepared, uploaded as a Google form link, and circulated to medical teachers through an electronic platform across the country The faculty responses were exported and analyzed using Microsoft Excel.

Results

Around 77.4% opined that making incremental changes to the old curriculum would have been better than the overhaul revision, and 85.6% have opined that input from more faculty must have been taken before implementing the new curriculum. Around 80% felt that the pace at which faculty are getting trained in the nodal/regional center is not adequate, and 75% of them believed that the faculty members are not adequate for preparatory work for CBME implementation. About 74.7% opined that framing specific learning objectives (SLOs) for all competencies is time-consuming.

Conclusion

It is the need of the hour for the curriculum to incorporate a systematic feedback mechanism built into the system. Despite the fact that many of the suggested changes are progressive, given the time and resource constraints, this can only be accomplished through the concerted and combined efforts of all those involved in medical education.

## Introduction

The medical education system all over the world is witnessing a paradigm shift from traditional methods of teaching to competency-based medical education (CBME) [1]. The new CBME was introduced in India with the 2019-2020 MBBS batch. The new curriculum has shifted its focus from knowledge-based medical training to CBME training. CBME co‑opts the national goals of “Health for All” by producing doctors who will be trained in providing holistic care, developing a scientific temper, and acting as ethical medical practitioners [2].

With the current curricular change, teachers are supposed to play a catalyst role in terms of moderating the different frameworks of CBME and motivating the stakeholders to be competent physicians. The new curriculum formulated different approaches like a foundation course, electives, integrated learning, and early clinical exposure for 360⁰ attainment of all the expected roles of Indian Medical Graduates (IMG). Despite the challenges, the charge of carrying it forward demands a distinctive intellectual and scholarly cast of medical educators [3].

A teacher plays multidimensional roles, namely being a facilitator, planner, manager, performance assessor, researcher, and mentor in addition to being a teacher [4-7]. Medical faculty with a passion for teaching is the process and product-oriented guides who can aid and guide the student’s career. Considering the fact that the whole concept of faculty development is still evolving, without formal preparation, the medical faculty may not be able to offer training for the students [3,8]. A well-planned faculty development strategy can address the deficiencies in the training of health professionals and escalate the possibility of successful implementation of CBME toward improved health outcomes [9,10].

This study aims to understand the faculty's views on the current CBME curriculum, the constraints they face in the new curriculum implementation, and their preparedness toward it.

## Materials and methods

This nationwide cross-sectional study was carried out among 297 teaching faculty representing 91 medical colleges across 20 states between February and July 2020. Prior institutional ethical committee approval was obtained (IHEC/04/2020). A face and content-validated structured questionnaire on the views of the newly implemented competency-based medical curriculum was prepared in the form of a five-point Likert scale. This self-developed questionnaire was validated by two internal experts and one external expert in medical education following which a pilot study was conducted among 40 teaching faculty (not part of the study). Informed consent was included in the questionnaire so that faculty who were unwilling to participate could opt out. There was an open-ended question section at the end to add their views if any. A nonprobability sampling method of volunteer opt-in sampling was done. The questionnaire was uploaded as a Google form link and was sent to medical teachers through social media platforms including WhatsApp, Telegram, Twitter, Facebook, etc. across the country. The anonymous faculty responses were exported and analyzed using Microsoft Excel. The response for each question was expressed as percentages.

We further explored the variations in results between the faculty from government and private medical colleges as well as between medical education unit (MEU)-trained (those who attended the Revised Basic Course workshop and obtained the certificate) and non-trained faculties using the subgroup analysis approach. The five-point Likert scale was scored as 5, 4, 3, 2, and 1 for strongly agree, agree, neutral, disagree, and strongly disagree, respectively. Data normality was ensured. We then obtained mean scores (with respective standard deviations) for each question under each domain for each subgroup. The scores were then compared using an independent sample t-test. Significance was taken at p < 0.05.

## Results

The proportion of faculty in government and private colleges constituted 2:3 (40% vs 60%). In both sectors, there was almost equal representation of the faculties who completed medical education training. Around 83% of the faculty accepted that there is a need to revise the traditional old curriculum. Around 77.4% opined that making stepwise incremental changes to the old curriculum would have been better than the overhaul revision. Nearly 47% felt that the new curriculum allows the students to learn at their own pace toward the attainment of prescribed competencies. About 85.6% have opined that input from more faculty must have been taken before implementing the new curriculum.

More than 85% of the participants have accepted that there is a need to update themselves on all the new concepts under CBME, and specific training is needed to equip themselves to train students on soft skills such as teamwork, interpersonal communication, and counseling. Around 80% felt that the pace at which faculty are getting trained in the nodal/regional center on the implementation of the new CBME curriculum is not adequate. Three-fourths of the participants opined that all the faculty may not demonstrate commitment to preparing case-based questions and facilitating small group teaching.

More than three-fourths of the participants believed that the faculty members are not adequate for preparing CBME implementation, designing early clinical exposure (ECE), integrating, and administering competency-based assessments and feedback. About 74.7% opined that framing specific learning objectives (SLOs) for all competencies is time-consuming, and 88.2% opined that sensitizing and training staff in CBME implementation strategies and development of standard evaluation systems need more time (Table 1).

**Table 1:** Descriptive analysis of faculty perceptions on the CBME curriculum CBME: Competency-based medical education; SLOs: Specific learning objectives; ECE: Early clinical exposure; SA: Strongly agree; A: Agree; N: Neutral; D: Disagree; SD: Strongly disagree.

S. No.	General Thoughts	Values in Percentage				
		SA	A	N	D	SD
1.	There is a need to revise the traditional (old) curriculum in undergraduate medical training.	46.1	37.0	8.4	5.4	3.0
2.	The new CBME curriculum serves the purpose of training the undergraduates better.	21.2	39.1	21.9	12.8	5.1
3.	Making small incremental changes to the existing curriculum (old) is better than overhaul revision.	33.3	44.1	11.1	7.4	4.0
4.	The new curriculum gives an opportunity for students to learn at their own pace toward the attainment of prescribed competencies.	13.8	33.0	27.3	17.8	8.1
5.	More faculty inputs could have been taken before implementing CBME.	62.0	23.6	10.4	2.0	2.0
Faculty Preparedness						
1.	We need to update ourselves on all the new concepts under CBME (like competencies, SLOs, and more).	45.8	41.4	9.1	1.7	2.0
2.	Specific training is needed to equip ourselves to effectively train students on soft skills such as teamwork, interpersonal communication, and counseling.	50.8	35.4	10.4	1.7	1.7
3.	The pace at which faculty are getting trained in the nodal/regional center is adequate (as these centers accept only two members per institution).	7.7	13.1	20.9	26.6	31.6
4.	I have downloaded and read the CBME document thoroughly.	31.6	43.4	11.4	6.1	7.4
5.	We cannot expect all faculty to demonstrate commitment to learn, prepare case-based questions, facilitate small group teaching, and motivate the students toward the new curriculum.	41.1	30.0	16.5	9.4	3.0
Time and Resource Constraints						
1.	Faculty members in departments are not adequate for CBME-related work and require a revamp in staff numbers.	68.0	16.2	11.4	2.0	2.4
2.	Designing ECE, integrating, and administering competency-based assessments and feedback are immensely challenging with limited academic staff.	33.0	42.4	18.2	5.1	1.3
3.	Framing SLOs for all competencies are time-consuming.	44.1	30.6	12.8	7.7	4.7
4.	Planning and implementing integrated modules are time-consuming considering the quantum of portions.	37.0	37.7	15.5	7.1	2.7
5.	Sensitizing and training staff in CBME implementation strategies and developing standard evaluation systems need more time.	50.2	38.0	5.7	2.7	3.4

**Figure 1:**
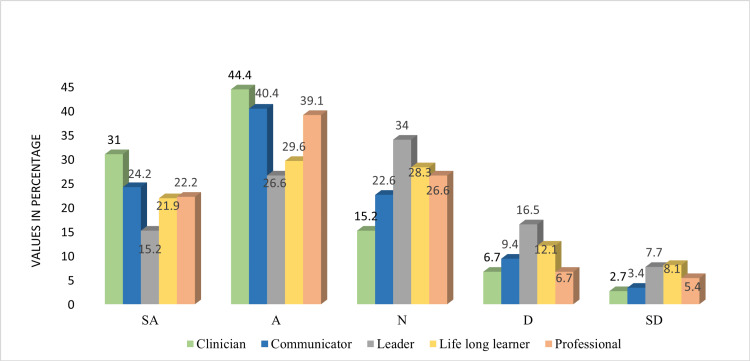
Attainment of the roles of Indian Medical Graduates SA: Strongly agree; A: Agree; N: Neutral; D: Disagree; SD: Strongly disagree.

The faculty responses for how far the students will progress in the absolute attainment of IMG roles and responsibilities like clinicians, communicators, leaders, lifelong learners, and professionals were 75.4%, 65.6%, 41.8%, 51.5%, and 61.3%, respectively (Figure 1).

We found no difference between the faculties of government and private medical colleges in terms of general thoughts, time, and resource constraints (p > 0.05). The mean scores were high, ranging between 3.09 and 4.31 among government faculty and between 3.34 and 4.48 among private medical college faculty. Private college faculty reported significantly higher scores for reading the CBME document thoroughly. Similarly, the mean scores of private college faculty were significantly higher for the attainment of roles of IMG in terms of clinicians, communicators, leaders, lifelong learners, and professionals (Figure 1, Table 2).

**Table 2:** Variation in the perceptions between government and private medical college faculties SLOs: Specific learning objectives; CBME: Competency-based medical education; ECE: Early clinical exposure; MD: Mean deviation; SD: Standard deviation.

S. No.	Parameters	Government Faculty		Private College Faculty		MD	T-value	P-value
		Mean	SD	Mean	SD			
General Thoughts								
1.	There is a need to revise the traditional (old) curriculum in undergraduate medical training.	4.04	1.13	4.26	0.94	0.21	-1.745	0.082
2.	The new CBME curriculum serves the purpose of training the undergraduates better.	3.43	1.13	3.68	1.1	0.25	1.906	0.06
3.	Making small incremental changes to the existing curriculum (old) is better than overhaul revision.	3.87	1.06	3.96	1.11	0.09	0.711	0.478
4.	The new curriculum gives an opportunity for students to learn at their own pace toward the attainment of prescribed competencies.	3.09	1.20	3.34	1.44	0.24	1.747	0.082
5.	More faculty inputs could have been taken before implementing CBME.	4.31	1.08	4.46	0.80	0.15	1.397	0.163
Faculty Preparedness								
1.	We need to update ourselves on all the new concepts under CBME (like competencies, SLOs, and more).	4.18	0.94	4.33	0.81	0.15	1.505	0.133
2.	Specific training is needed to equip ourselves to effectively train students on soft skills such as teamwork, interpersonal communication, and counseling.	4.20	0.99	4.38	0.79	0.18	1.724	0.086
3.	The pace at which faculty are getting trained in the nodal/regional center is adequate (as these centers accept only two members per institution).	2.32	1.26	2.38	1.28	0.05	0.344	0.731
4.	I have downloaded and read the CBME document thoroughly.	3.63	1.22	3.97	1.15	0.33	2.432	0.016
5.	We cannot expect all faculty to demonstrate commitment to learn, prepare case-based questions, facilitate small group teaching, and motivate the students toward the new curriculum.	3.84	1.24	3.98	1.13	0.15	1.05	0.294
Time and Resource Constraints								
1.	Faculty members in departments are not adequate for CBME-related work and require a revamp in staff numbers.	4.29	1.28	4.48	0.88	0.19	1.52	0.128
2.	Designing ECE, integrating, and administering competency-based assessments and feedback are immensely challenging with limited academic staff.	3.73	1.16	3.79	1.14	0.05	0.41	0.686
3.	Framing SLOs for all competencies is time-consuming.	3.94	1.22	4.04	1.14	0.10	0.758	0.449
4.	Planning and implementing integrated modules are time-consuming, considering the quantum of portions.	3.89	1.21	3.99	1	0.1	0.782	0.435
5.	Sensitizing and training staff in CBME implementation strategies and developing standard evaluation systems need more time.	4.228	1.05	4.287	0.97	0.05	0.5	0.617
Attainment of Roles of Indian Medical Graduates								
1.	Clinician	3.68	1.19	4.03	0.99	0.36	2.842	0.005
2.	Communicator	3.812	1.17	3.80	1.06	0.29	2.242	0.026
3.	Leader	2.93	1.24	3.38	1.14	0.46	3.282	0.001
4.	Lifelong learner	3.17	1.28	3.57	1.20	0.40	2.781	0.006
5.	Professional	3.45	1.16	3.71	1.14	0.26	1.934	0.054

The study findings show that the faculty trained in MEU report significantly higher mean scores requiring specific training to equip themselves to effectively train students on soft skills (p < 0.05). Faculty not trained in MEU felt that the pace at which faculties are being trained in the nodal/regional centers is inadequate (p < 0.05). Importantly, a significant number of faculty trained in MEU reported that departments are not adequate for CBME-related work and require a revamp in staff numbers in comparison to faculty not trained in MEU (p < 0.05) (Table 3).

**Table 3:** Variation in perceptions between MEU-trained and MEU-nontrained medical college faculties MEU: Medical education unit; SLOs: Specific learning objectives; CBME: Competency-based medical education; ECE: Early clinical exposure; MD: Mean deviation; SD: Standard deviation.

S. No.	Parameters	MEU-Trained		Not MEU-Trained		MD	T-value	P-value
		Mean	SD	Mean	SD			
General Thoughts								
1	There is a need to revise the traditional (old) curriculum in undergraduate medical training.	4.16	1.07	4.19	0.97	0.03	-0.301	0.763
2	The new CBME curriculum serves the purpose of training the undergraduates better.	3.58	1.19	3.58	1.03	0.0005	0.004	1
3	Making small incremental changes to the existing curriculum (old) is better than overhaul revision.	3.93	1.14	3.91	1.03	0.02	0.179	0.85
4	The new curriculum gives an opportunity for students to learn at their own pace toward the attainment of prescribed competencies.	3.22	1.23	3.23	1.11	0.03	-0.267	0.79
5	More faculty inputs could have been taken before implementing CBME.	4.37	0.96	4.43	0.89	0.06	-0.646	0.52
Faculty Preparedness								
1	We need to update ourselves on all the new concepts under CBME (like competencies, SLOs, and more).	4.28	0.91	4.26	0.82	0.01	0.177	0.86
2	Specific training is needed to equip ourselves to effectively train students on soft skills such as teamwork, interpersonal communication, and counseling.	4.4	0.9	4.19	0.87	0.21	2.063	0.04
3	The pace at which faculty are getting trained in the nodal/regional center is adequate (as these centers accept only two members per institution).	2.58	1.26	2.1	1.23	0.49	3.439	0.001
4	I have downloaded and read the CBME document thoroughly.	3.86	1.29	3.81	1.08	0.05	0.346	0.73
5	We cannot expect all faculty to demonstrate commitment to learn, prepare case-based questions, facilitate small group teaching, and motivate the students toward the new curriculum.	3.97	1.2	3.87	1.16	0.1	0.753	0.45
Time and Resource Constraints								
1	Faculty members in departments are not adequate for CBME-related work and require a revamp in staff numbers.	4.51	1.03	4.27	1.1	0.24	1.936	0.05
2	Designing ECE, integrating, and administering competency-based assessments and feedback are immensely challenging with limited academic staff.	3.81	1.11	3.7	1.2	0.11	0.82	0.41
3	Framing SLOs for all competencies are time-consuming.	4.04	1.17	3.95	1.18	0.08	0.646	0.52
4	Planning and implementing integrated modules are time-consuming, considering the quantum of portions.	3.99	1.1	3.91	1.08	0.08	0.607	0.54
5	Sensitizing and training staff in CBME implementation strategies and developing standard evaluation systems need more time.	4.27	1.06	4.25	0.92	0.02	0.166	0.87
Attainment of Roles of Indian Medical Graduates								
1	Clinician	3.97	1.06	3.78	1.12	0.2	1.573	0.12
2	Communicator	3.66	1.13	3.71	1.1	0.04	-0.374	0.71
3	Leader	3.27	1.21	3.1	1.19	0.17	1.202	0.23
4	Lifelong learner	3.41	1.29	3.41	1.2	0.002	0.015	1
5	Professional	3.55	1.2	3.66	1.1	0.11	-0.786	0.43

## Discussion

This cross-sectional study attempts to highlight the views of faculty on the new CBME curriculum, faculty preparedness, and the issues related to the delivery of the new curriculum.

More than four-fifths of the participants felt that there was a need to revise the new curriculum and that the new CBME curriculum helps in creating the Ideal Indian Medical Graduate as defined by the National Medical Commission. In their study of 105 participants of the medical fraternity, Narwane et al. observed nearly similar results. The traditional curriculum was subject-centered and time-based, which places its emphasis more on knowledge and to some extent on skill training, while the other important domains like attitude, communication, and clinical skills were not given much importance. The individualized learning facilitated through CBME will ensure that all the competencies are being met for each stage, thereby facilitating the production of a successful IMG [11,12].

While we intend to improve the quality of new medical educational interventions, capacity building of faculty and skill upgradation assume the top priority. More than 80% felt that they need to update themselves on the new concepts of the curriculum, and hence they need a qualitative faculty development program for all the faculty at the earliest to facilitate smooth operationalization of the CBME. Similar views are being discussed by many authors. As the faculty play a key role in the successful implementation of CBME, a more elaborative prioritized longitudinal faculty development program with self-reinforcing workshops for the faculties at regular intervals may provide an opportunity for behavior review, practice, reflection, and reinforcement of learned actions must be conducted in higher frequencies than the current one so that more resource faculty from new/existing colleges can be effectively trained in the nodal center who in turn will train their colleagues to bring the change in their institution [11-15]. In the present study, faculty who had not been trained in MEU expressed concerns about the pace at which they were trained in the nodal and regional centers as these centers accept only two members per institution. Also, a mere three days of curriculum implementation support program (CISP) cannot impart the expected skills as the teachers were not exposed at all during their own undergraduate training [1,16]. The MEU-trained faculty reported the need for additional specific training to equip themselves to effectively train students on soft skills such as teamwork, interpersonal communication, and counseling, thereby signifying the inadequacy of the existing training framework and institutional infrastructure.

More than three-fourths of the participants have opined that there are already minimal faculty members in the departments as per the current norms. Faculty trained in MEU expressed significantly greater concerns regarding the deficiency of faculty in various departments; this signifies that MEU-trained faculty feel burdened with the number of new activities to be undertaken with the introduction of CBME. The faculties are overburdened, and they do not have quality time for implementation of components mentioned in the CBME curriculum like framing SLOs; planning integration; designing; conducting ECE and assessments; delivering feedback; conducting attitude, ethics, and communication module (AETCOM) and small group discussion; etc. Many authors have also put forth the same discussion stating that a limited number of staff with more workload makes them exhausted/disinterested in the implementation activities of the new curriculum apart from their regular routine [1,16-18].

Nealy half of the respondents have opined that the new curriculum has given less scope for a student to be a leader and lifelong learner. The new curriculum has given many scopes for the development of these competencies. Lifelong learning is a professional competency that must be fostered in medical graduates. Four attributes of a lifelong learner, namely metacognition, self-directed learning, self-monitoring, and having a reflective attitude, can be cultivated by explicitly using instructional methodologies like problem-based learning, e-learning, reciprocal teachings, portfolios, reflections, and knowledge maps. Once these lifelong learning attributes are inculcated by the medical graduate, the medical practitioners will always be "current" in medical knowledge and skills and will be able to give better medical care [19]. The AETCOM module when implemented well will ensure the development of leadership qualities.

This study has a few limitations. First, being an electronic survey, the sampling method could not account for the volunteer bias. Second, we did not refine the questionnaire by performing factor analysis. However, we performed face and content validity of the questionnaire to ensure quality. Third, the study did not capture the sociodemographic characteristics of the study participants based on which a subgroup analysis could have been performed.

It is the need of the hour to explore the perceived and experienced challenges in the implementation of CBME using a nationally representative sample with regional-level disaggregated results and recommendations. It is equally important to sensitize and brainstorm with the stakeholders, namely students, teachers, and administrative authorities, with an emphasis on handling these challenges for the smooth implementation of the curriculum. This should go hand in hand with faculty training with different teaching, learning, and assessment methods.

## Conclusions

Capacity building of the faculty is the major issue that needs to be prioritized in the implementation of the new CBME curriculum. Lack of sufficient qualified staff threatens student skill-based learning and reduces the effectiveness of teaching-learning. The pandemic era has further delayed effective implementation. A qualitative and robust faculty development program at a faster pace with an increase in the number of committed faculty members in each department will be a sustainable approach toward the successful implementation of the new CBME in India.

## References

[1] Telang A, Rathod S, Supe A, Nebhinani N, Mathai S (2017). Faculty views on competency- based medical education during mentoring and learning web sessions: an observational study. J Educ Technol Health Sci.

[2] Medical Council of India (2018). Competency based undergraduate curriculum. https://www.nmc.org.in/information-desk/for-colleges/ug-curriculum/.

[3] Schofield SJ, Bradley S, Macrae C, Nathwani D, Dent J (2010). How we encourage faculty development. Med Teach.

[4] Chacko TV (2014). Moving toward competency-based education: challenges and the way forward. Arch Med Health Sci.

[5] Boursicot K, Kemp S, Wilkinson T, Findyartini A, Canning C, Cilliers F, Fuller R (2021). Performance assessment: consensus statement and recommendations from the 2020 Ottawa Conference. Med Teach.

[6] Srivastava TK, Waghmare LS, Rawekar A, Mishra VP (2016). Fostering educational research among medical teachers: evaluation of a faculty development program in India. J Clin Diagn Res.

[7] Wang Y, Kretschmer RE, Hartman MC (2010). Teacher-as-researcher: theory-into-practice. Am Ann Deaf.

[8] Stenfors-Hayes T, Hult H, Dahlgren LO (2011). What does it mean to be a mentor in medical education?. Med Teach.

[9] Bansal P, Supe A, Sahoo S (2018). Faculty development for competency based medical education: Global, national and regional perspectives: faculty development for competency based medical education. Natl J Integr Res Med.

[10] Frei E, Stamm M, Buddeberg-Fischer B (2010). Mentoring programs for medical students - a review of the PubMed literature 2000-2008. BMC Med Educ.

[11] Narwane S, Anikhindi A, Mahavarkar V (2021). Study of perceptions of competency based medical education reforms in India. Pravara Med Rev.

[12] Rajashree R, Chandrashekar DM (2020). Competency- based medical education in India: a work in progress. Indian J Physiol Pharmacol.

[13] Nagarala MS, Devi RML (2021). Faculty development programs for implementing competency based medical education in India: challenges and opportunities. Int J Community Med Public Health.

[14] Elif I (2021). Evaluation of competency based medical education curriculum. Int J Prog Edu.

[15] Modi JN, Gupta P, Singh T (2015). Competency-based medical education, entrustment and assessment. Indian Pediatr.

[16] Sharma R, Bakshi H, Kumar P (2019). Competency-based undergraduate curriculum: a critical view. Indian J Community Med.

[17] Shah N, Desai C, Jorwekar G, Badyal D, Singh T (2016). Competency-based medical education: an overview and application in pharmacology. Indian J Pharmacol.

[18] Kaushik A, Jaiswal A, Singh A K, Rizvi G (2022). Challenges to new undergraduate medical curriculum due to COVID-19pandemic and possible solution in India. Med J DY Patil Vidyapeeth.

[19] Mahajan R, Badyal DK, Gupta P, Singh T (2016). Cultivating lifelong learning skills during graduate medical training. Indian Pediatr.

